# ‘2A‐Like’ Signal Sequences Mediating Translational Recoding: A Novel Form of Dual Protein Targeting

**DOI:** 10.1111/tra.12411

**Published:** 2016-06-02

**Authors:** Claire Roulston, Garry A. Luke, Pablo de Felipe, Lin Ruan, Jonathan Cope, John Nicholson, Andriy Sukhodub, Jens Tilsner, Martin D. Ryan

**Affiliations:** ^1^ Biomolecular Sciences BuildingUniversity of St AndrewsNorth Haugh, St AndrewsFifeKY16 9STScotlandUK; ^2^ Spanish Medicines Agency (AEMPS), Parque Empresarial “Las Mercedes”Campezo 1 – Edificio 828022MadridSpain; ^3^ Oakland InnovationHarston Mill, HarstonCambridgeCB22 7GGUK; ^4^ James Hutton InstituteInvergowrieDundeeDD2 5DAUK

**Keywords:** 2A, dual protein targeting, secretory pathway, signal sequence, translational recoding

## Abstract

We report the initial characterization of an N‐terminal oligopeptide ‘2A‐like’ sequence that is able to function both as a signal sequence and as a translational recoding element. Owing to this translational recoding activity, two forms of nascent polypeptide are synthesized: (i) when 2A‐mediated translational recoding has not occurred: the nascent polypeptide is fused to the 2A‐like N‐terminal signal sequence and the fusion translation product is targeted to the exocytic pathway, and, (ii) a translation product where 2A‐mediated translational recoding has occurred: the 2A‐like signal sequence is synthesized as a separate translation product and, therefore, the nascent (downstream) polypeptide lacks the 2A‐like signal sequence and is localized to the cytoplasm. This type of dual‐functional signal sequence results, therefore, in the partitioning of the translation products between the two sub‐cellular sites and represents a newly described form of dual protein targeting.

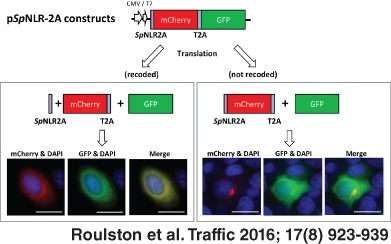

The function of the 2A oligopeptide sequence (18aa) was first characterized from the positive‐stranded RNA picornavirus Foot‐and‐Mouth Disease Virus (FMDV). FMDV 2A was shown to mediate a co‐translational ‘cleavage’ between the upstream (capsid proteins) and downstream (RNA replication proteins) domains of the FMDV polyprotein (Figure 1A; Refs 1, 2, 3, 4, 5). Active 2A sequences were characterized in the genomes of viruses from other genera of the picornaviruses, plus ‘2A‐like’ sequences found within the genomes of a range of different RNA viruses and non‐LTR retrotransposons 6, 7, 8, 9, 10. These 2A/‘2A‐like’ oligopeptide sequences were shown to mediate a translational ‘recoding’ event referred‐to as ‘ribosome skipping’, ‘stop carry‐on’ or ‘stop‐go’ translation 11, 12, 13, 14, 15, 16. Briefly, when an elongating ribosome encounters 2A, it ‘skips’ the synthesis of a specific glycyl‐prolyl peptide bond. Although no stop codon is involved, eukaryotic translation release (termination) factors 1 and 3 (eRF1/eRF3) are proposed to release the nascent protein from the ribosome, thereby forming the C‐terminus of 2A (Figure 2). In support of this model, eRF3 was recently shown to mediate termination of translation of yeast ribosomes stalled on polylysine tracts 17, although other recent analyses using re‐constituted translation systems did not support the involvement of eRFs 1 and 3 18. Our model of this translational recoding event predicts that three alternative outcomes arise; having synthesized sequences upstream of 2A, ribosomes either (i) resume translation of the downstream sequences, (ii) translation is terminated at that point, or, (iii) that no translational recoding occurs: the glycyl‐prolyl peptide bond is formed and the protein is synthesized in the normal manner. The ratio of these translation products is dependent upon the specific 2A‐like sequence in question – a model supported by the observations from many other laboratories that have used 2A for biomedical or biotechnological protein co‐expression applications 19.

**Figure 1:**
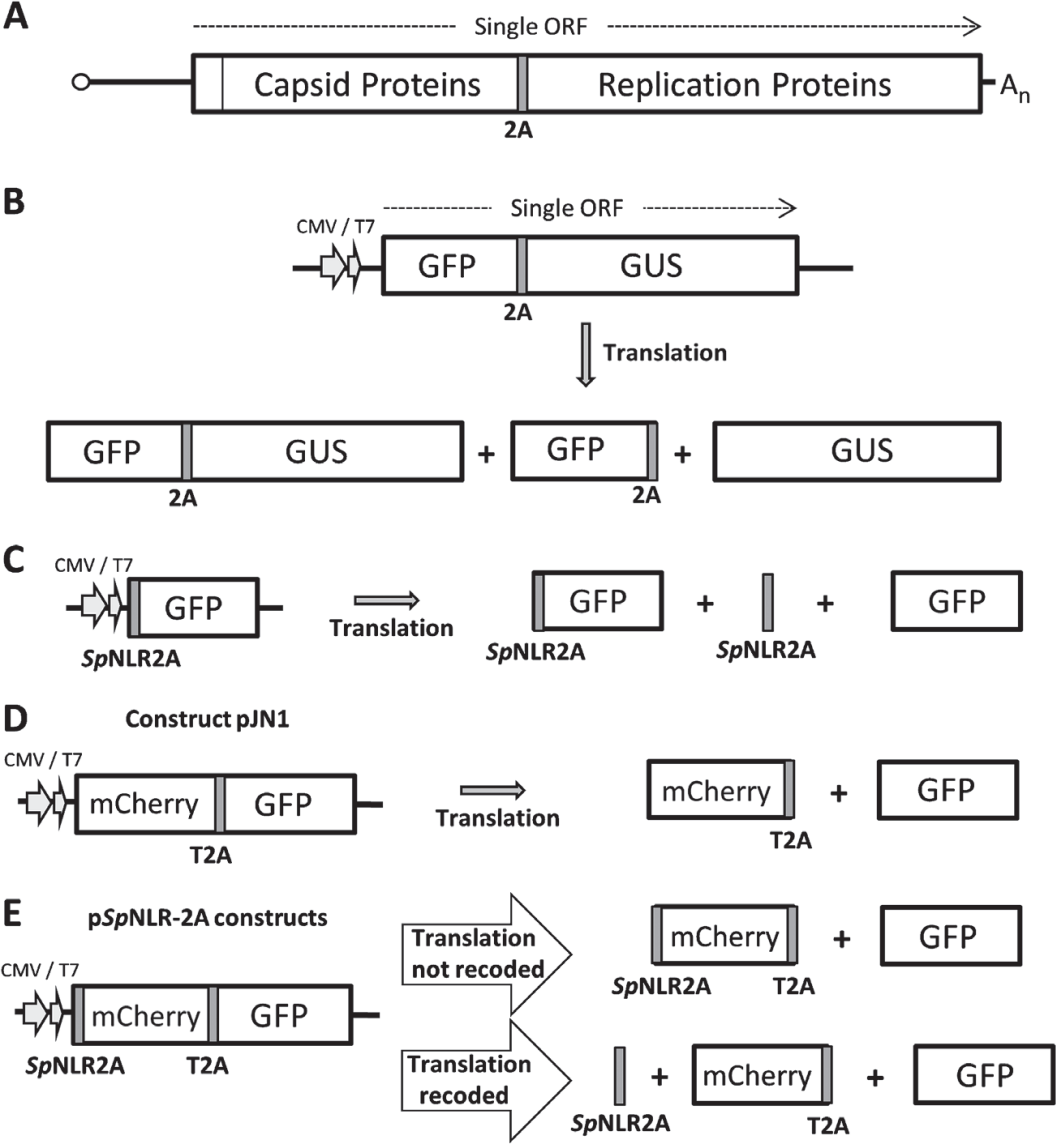
**Occurrence of 2A and plasmid constructs.** A) 2A‐mediated translational recoding serves to separate the capsid proteins and domains comprising replicative proteins of the FMDV polyprotein. B) Plasmids encoding the [GFP‐2A‐GUS] artificial polyprotein system were used to analyze 2A recoding activities by the quantification of the ‘uncleaved’ [GFP‐2A‐GUS] and ‘cleaved’ [GFP‐2A] plus GUS translation products. C) The wild‐type SpNLR2A6 sequence and mutated forms were fused to the N‐terminus of GFP for analyses of recoding activities by comparison of the levels of the [SpNLR2A6^WT^‐GFP] and GFP products. D) Plasmid pJN1 was used as a control for the sub‐cellular localisation studies: the T2A sequence ‘cleaves’ to produce [mCherry‐T2A] and GFP. Neither protein has a signal sequence and both colocalize within the cytoplasm. E) SpNLR2A sequences were fused to the N‐terminus of mCherry: the signal sequence activities were determined by the extent of colocalization with (cytoplasmic) GFP.

**Figure 2:**
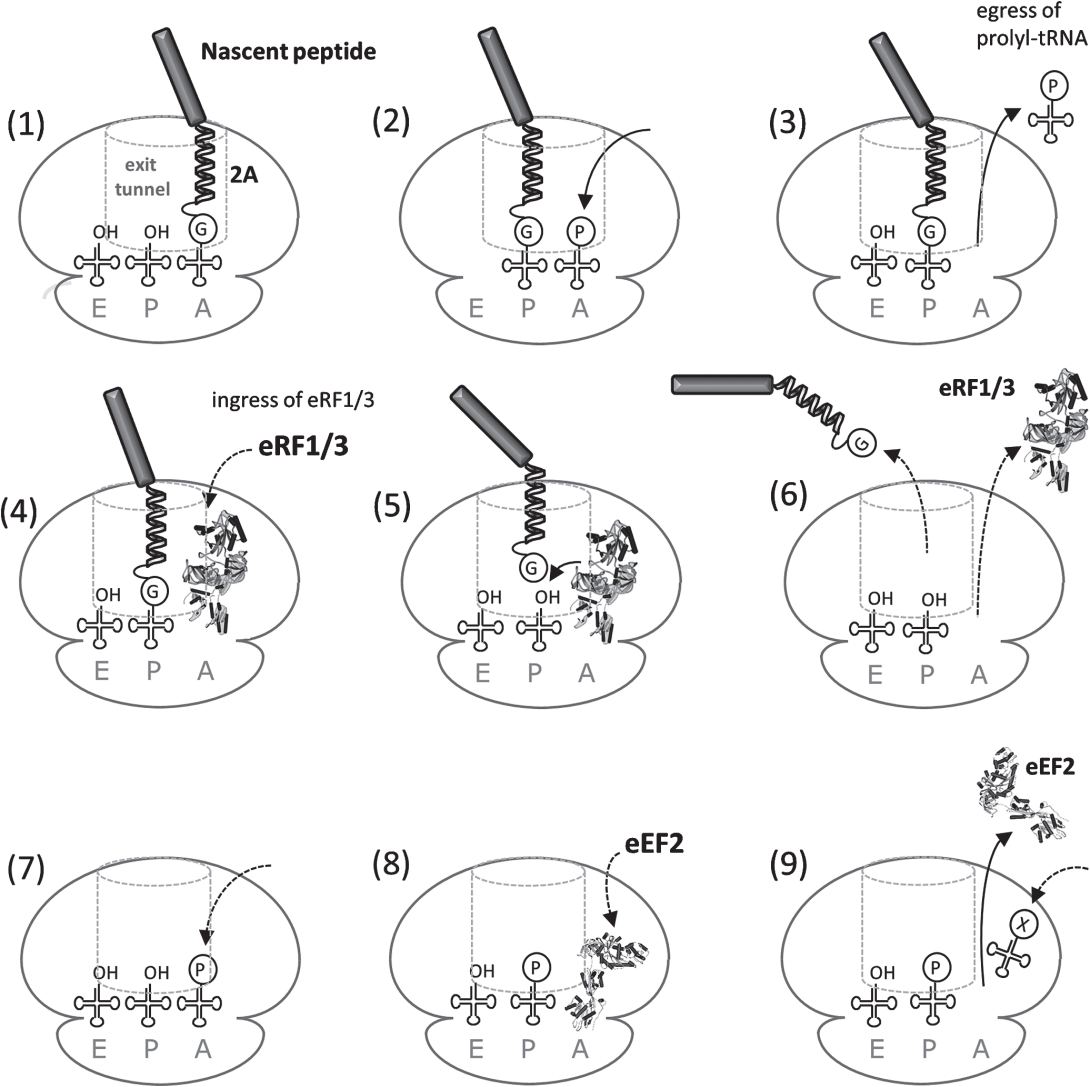
**Model of 2A‐mediated translational ‘recoding’.** When the nascent peptide upstream of 2A is emerging from the ribosome, 2A is positioned in the ribosome exit tunnel (step 1). The nascent peptide‐2A and glycyl‐tRNA is translocated from the A‐ to P‐site and prolyl‐tRNA enters the A‐site (step 2). The nascent 2A peptide interacts with the exit tunnel of the ribosome such that the C‐terminal portion (−ESNPG‐) is sterically constrained within the peptidyl‐transferase centre (PTC) of the ribosome. Nucleophilic attack of the ester linkage between 2A and tRNA^gly^ by prolyl‐tRNA in the A‐site is inhibited – effectively stalling, or pausing, translation. The failure to form a new peptide bond leads to dissociation of prolyl‐tRNA from the A‐site of the ribosome (step 3), required for entry of release factors into the A‐site (step 4). We have shown that this block is relieved by the action of translation release factors eRF1 and eRF3, hydrolyzing the ester linkage (step 5) and releasing the nascent protein (step 6). eRF1 leaves the complex, eRF3 being involved in this process (step 6). Two, mutually exclusive, outcomes may then arise: (i) translation terminates at the C‐terminus of 2A, or, (ii) prolyl‐tRNA (re)enters the A‐site (step 7), is translocated by eukaryotic elongation factor 2 (eEF2) from the A‐ to the P‐site (step 8), allowing the next amino‐acyl tRNA to enter the A‐site to permit the synthesis of the sequences downstream of 2A (step 9).

Our analyses of this recoding event were based upon the creation of an artificial polyprotein system comprising green fluorescent protein (GFP; stop codon removed) linked, *via* the 2A‐like sequence being analysed, to β‐glucuronidase (GUS; initiation codon removed) to create a single open reading frame (ORF; [GFP‐2A‐GUS]; Figure 1B). The translation products from these experiments are (i) the [GFP‐2A‐GUS] fusion protein, (ii) [GFP‐2A] – 2A remains as a C‐terminal extension of the upstream protein, and (iii) GUS. For the most active 2A/2A‐like sequences, the major products (90–99%) are [GFP‐2A] and GUS. The full‐length translation product [GFP‐2A‐GUS] is, however, also observed – particularly for 2A/2A‐like sequences with lower recoding activities 8, 9. In some cases the biological function of 2A lies in very high – essentially complete – ‘cleavage’ to generate the individual, discrete, translation products, while in other cases the biological function of 2A appears to be the generation of a mixture of both ‘cleaved’ (translation ‘recoded’) and ‘uncleaved’ forms (translation not recoded) – the proportions of which varies among different 2A‐like sequences. For simplicity, the terms ‘cleaved’/‘uncleaved’ will be used below.

In all the cases outlined above, the 2A/2A‐like sequences are present within virus or ‘virus‐like’ – non‐LTR (long terminal repeat) retrotransposon genomes and encoded at internal sites within proteins/polyproteins. A motif (−D[V/I]ExNPGP−) is conserved at the C‐terminus of all 2A/2A‐like sequences: this motif must, however, be accompanied by an appropriate, but much less conserved, upstream context for the 2A/2A‐like sequence to function as a translational recoding element. Probing databases with this C‐terminal motif identified numerous 2A‐like sequences in non‐LTR retrotransposons within the genome of *Trypanosoma cruzi* (and other trypanosome species, Ref 7) and, latterly, in a range of other species from different families/genera 9. For one of these species, the purple sea urchin *Strongylocentrotus purpuratus*, 2A‐like sequences were also identified in genes involved in the innate immune system. *S. purpuratus* has a large repertoire of immune‐related genes that recognize pathogen‐associated molecular patterns 20, 21. The NOD‐like receptor proteins (NLR; nucleotide‐binding domain and leucine‐rich repeat) gene family encode proteins with a common architecture: an N‐terminal effector‐binding (DEATH) domain, a NACHT nucleotide‐binding domain and a C‐terminal domain comprising leucine‐rich repeats. These NLR proteins localize within the cytoplasm whereas a similar family of pattern recognition receptors, the toll‐like receptors, possess transmembrane domains and localize to the plasma or endosomal membranes (reviewed in 22, 23, 24, 25, 26). Bioinformatic analyses showed that ∼35% (85/241) of these *S. purpuratus* NLR proteins contain 2A‐like sequences: surprisingly not as internal features, but either at their N‐termini, or, within the N‐terminal region. The *S. purpuratus* NLR N‐terminal 2A‐like sequences (*Sp*NLR2As) we have identified are shown in Table 1. Furthermore, bioinformatic analyses also showed a high proportion of these *Sp*NLR2As were predicted to function as co‐translational signal sequences. Two questions arose immediately; (i) are these 2A‐like sequences active in translational recoding? and (ii) if they remain fused to the downstream sequences, could they indeed function as a co‐translational signal sequence? Since these *Sp*NLR2As were N‐terminal features, we adopted the same strategy of using an artificial polyprotein reporter system, but adapted such that we could use the same type of construct as the basis for the analyses of both the recoding activity *and* the effect of these *Sp*NLR2As on protein sub‐cellular localization. Since the function of signal sequences is known to be retained across diverse eukaryotic cell‐types 27, for pragmatic reasons we studied these potential echinoderm signal sequences using mammalian cells. To extend the scope of any potential biotechnological uses of these sequences we also examined ‘cleavage’ and sub‐cellular protein targeting within plant cells.

**Table 1:**
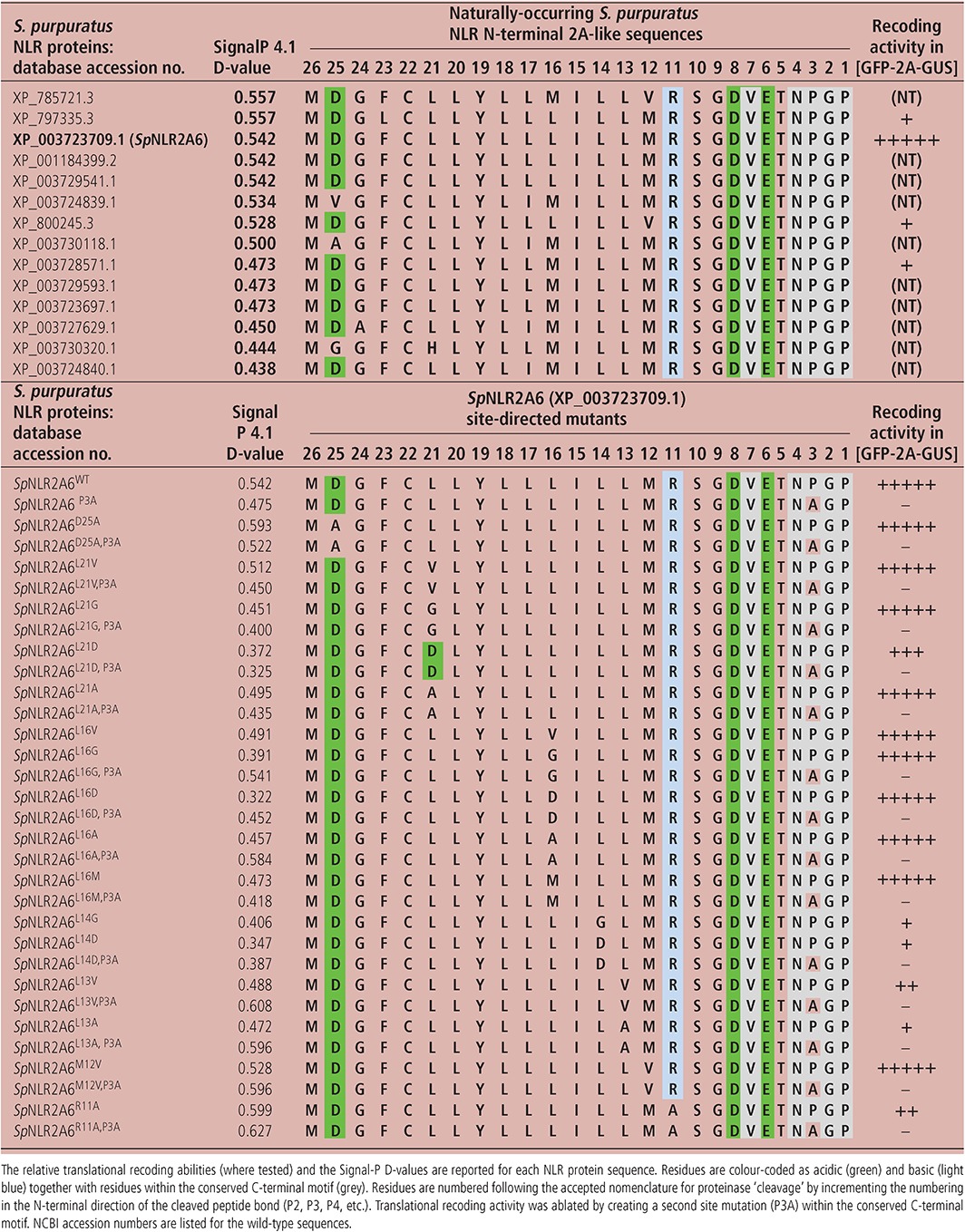
S. purpuratus N‐terminal NLR 2A‐like sequences: wild‐type sequences and site‐directed mutants

Our data show that this N‐terminal form of 2A possesses both translational recoding and signal sequence activities in mammalian and plant cells. We propose that these translational recoding signal sequences represent a newly characterized form of dual protein targeting: in the proportion of cases where a peptide bond is formed between the *Sp*NLR2A signal sequence and the downstream protein (translation not recoded), then the fusion protein is targeted to the exocytic pathway. Where the peptide bond is not formed (‘skipped’) by the translational recoding activity of the *Sp*NLR2A, then the signal sequence and the downstream protein emerge from the ribosome as individual, discrete, translation products. While we have not investigated the fate of the oligopeptide *Sp*NLR2A signal sequence [interaction with signal recognition particle (SRP) or degradation?], we show that lacking an N‐terminal signal sequence the downstream protein localizes to the cytoplasm – taken together, a novel form of dual protein targeting.

## Results

### Bioinformatic analyses

Sequence databases were probed with the conserved –D(V/I)ExNPGP‐ motif using BLASTp (initially at the Baylor College of Medicine, latterly at NCBI). *S. purpuratus* sequences encoding 2A‐like sequences could be divided into two main categories: non‐LTR retrotransposons 9 and NLR proteins. Probing the NCBI database for NLR sequences (using the NACHT domain from NP_001120727.1 as a probe) shows that the early figure for NLR proteins encoded within the *S. purpuratus* genome (203 NLR proteins; Ref 20) was reduced to 87 entries in NCBI, 14 of which possess a 26aa 2A‐like sequence at their N‐termini (Table 1). These sequences were then analysed using SignalP 4.1 28 for signal peptide prediction: the D‐cutoff values are shown in Table 1. Of the 14 such sequences analysed, 12 were predicted to function as co‐translational signal sequences (D‐cutoff values ≥ 0.45). The algorithm also predicted a signal peptidase complex cleavage site towards the C‐terminus of the *Sp*NLR2A6^WT^ (−VET**^↓^**NPG‐) and *Sp*NLR2A6^P3A^ (−VET**^↓^**NAG‐) sequences.

### Functional assays

Since co‐translational signal sequences are known to function across kingdoms 27, we chose to analyze both the translational recoding and protein targeting functions of these *Sp*NLR2As in an immortalized cell type (mammalian HeLa) readily available to us, as no immortalized echinoderm cell‐lines were available. The tandem CMV and T7 promoters in the pCDNA plasmid backbone used for DNA constructions facilitated analyses by both programming a coupled *in vitro* transcription/translation system (T7 promoter), or, be used to direct transcription following transfection of mammalian cells (CMV promoter) to study cellular expression and protein sub‐cellular localization (Figure 1C). Our control construct (pJN1; Figure 1D) comprised a single ORF encoding [mCherry‐T2A‐GFP] to which the *Sp*NLR2A sequences were added as N‐terminal fusions. The highly efficient T2A sequence ‘cleaves’ GFP as a discrete product, providing an internal control for the cell expression studies since, in the absence of a signal sequence, both the mCherry and GFP localize to the cytoplasm. The inclusion of a translational recoding signal sequence that does not ‘cleave’ and remains fused at the N‐terminus would alter the sub‐cellular localization of the translation products produced from the p*Sp*NLR2A6^WT^‐mCherry‐T2A‐GFP plasmid constructs (Figure 1E) such that the [*Sp*NLR2A6^WT^‐mCherry‐T2A] would now enter the exocytic pathway while the GFP would localize to the cytoplasm 29. ‘Cleavage’ of the signal sequence would produce mCherry and GFP colocalization in the cytoplasm. For expression within plant cells (*Nicotiana benthamiana*), the inserts were transferred to a Tobacco mosaic virus (TMV)‐based over‐expression vector to produce intense fluorescence and facilitate clear determination of sub‐cellular localization 30.

We chose to base our analyses upon the *Sp*NLR2A sequence from the database entry XP_003723709.1 (designated *Sp*NLR2A6) which scored highly in the SignalP4.1 analyses (Table 1). As a control for both translational recoding and sub‐cellular localization experiments, we created a single point mutant (P3A) which we have previously shown to ablate recoding activity 6.

### Functional assay: translational recoding activities

**Figure 3:**
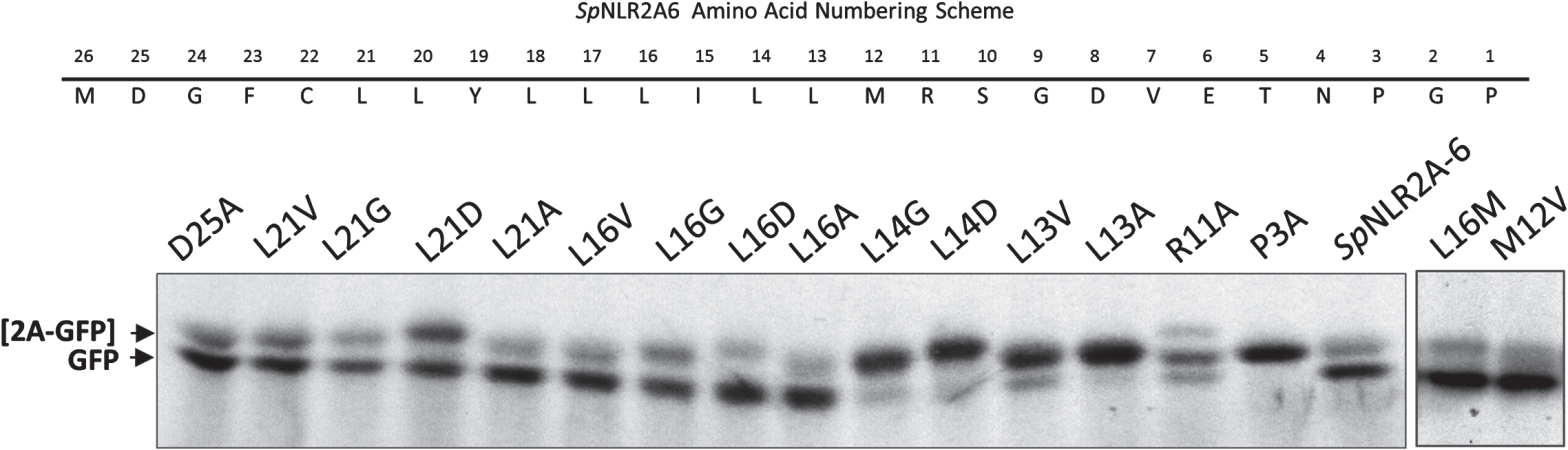
**In vitro coupled transcription/translation analyses.** SDS–PAGE analyses of the wild‐type sequence is shown, together with the residue numbering system. Coupled transcription/translation systems were programmed with plasmid DNA encoding the point mutations as indicated. The distribution of radiolabel was visualized by autoradiography. Note: All doubly‐mutated sequences bearing the P3A mutation were recoding inactive (data not shown).

To analyze the translation recoding activities of the *Sp*NLR2A6 sequences, plasmid constructs were used to program cell‐free coupled transcription/translation systems. Such systems lack any ER‐derived membranes, so only show *de novo* protein synthesis without any ‘processing’ of signal peptides. The expected translation products from the various *Sp*NLR2A6 constructs were the ‘uncleaved’ form [*Sp*NLR2A6^P3A^‐GFP] (29.9 kDa) together with the GFP ‘cleavage’ product (∼27 kDa) (Figures 1C and 3). The recoding activity of the wild‐type *Sp*NLR2A6 sequence was high (qualitatively estimated at ∼80%), while the control mutant, *Sp*NLR2A6^P3A^, was completely inactive for translational recoding. Mutations within the sequence (R11A, L13V, L13A, L14G, L14D) proximal to the conserved –D(V/I)ExNPGP‐ motif, but within a short tract of long side‐chain hydrophobic residues present within many 2A‐like sequences 8, substantially reduced the recoding activity. Additional mutations more distal (D25A, L21V, L21D) reduced recoding activity to a lesser extent. Other mutations (L21G, L21A, L16V, L16G and L16D) had little, or no, effect on recoding (Figure 3). The two naturally‐occurring sequences that varied from *Sp*NLR2A6 (L16M, M12V) were as active as the wild‐type *Sp*NLR2A6 sequence. These data are consistent with our previous analyses of site‐directed mutants 8, but suggest that within the range of naturally‐occurring *Sp*NLR2A sequences (Table 1) a range of translation recoding (and, therefore, a range in the partitioning of translation products between different sub‐cellular sites) would be observed.

### Protein localization: expression in mammalian cells

**Figure 4:**
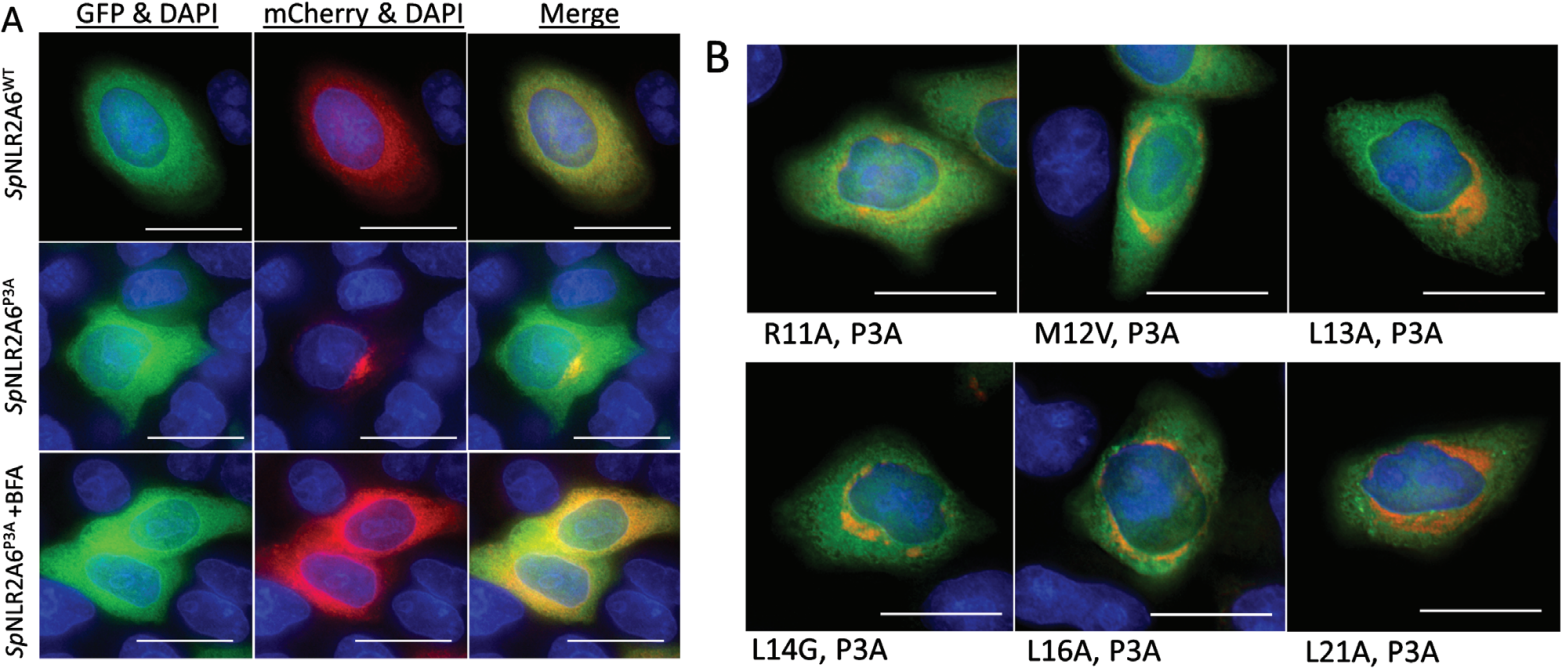
**In vivo expression.** Mammalian (HeLa) cells were transfected with plasmid DNA as indicated and stained with DAPI (scale bar = 15 μM). Both translation products from pSpNLR2A6^WT^‐mCherry‐T2A‐GFP localized to the cytoplasm (Panel A: upper images), whereas in the case of cells transfected with plasmid pSpNLR2A6^P3A^‐mCherry‐T2A‐GFP, fluorescence from GFP was localized to the cytoplasm while fluorescence from mCherry predominantly localized to the Golgi apparatus (Panel A: centre images). Brefeldin A treatment of cells transfected with plasmid pSpNLR2A6^P3A^‐mCherry‐T2A‐GFP collapsed the mCherry fluorescence back onto the endoplasmic reticulum (Panel A: lower images). Analyses of all of the doubly‐mutated forms (all comprising the P3A mutation) showed the same pattern of protein localization as for pSpNLR2A6^P3A^‐mCherry‐T2A‐GFP (Panel B).

Transfection of HeLa cells with the reporter plasmid encoding the wild‐type *Sp*NLR2A6 signal sequence (p*Sp*NLR2A6^WT^‐mCherry‐T2A‐GFP, Figure 1D), highly active in translational recoding and, therefore, ‘cleavage’ of the signal sequence, showed both the major cleavage products [mCherry‐T2A] and GFP to localize to the cytoplasm (Figure 4A, upper panel). Transfection of HeLa cells with the plasmid encoding the mutant *Sp*NLR2A6 signal sequence (p*Sp*NLR2A6^P3A^‐mCherry‐T2A‐GFP), inactive for translational recoding and, therefore, the p*Sp*NLR2A6^P3A^ signal sequence remaining fused to mCherry, showed that while the GFP cleavage product localized to the cytoplasm, the [*Sp*NLR2A6^P3A^‐mCherry‐T2A] now localized primarily to the Golgi apparatus (Figure 4A, middle panel), confirmed by brefeldin A treatment (BFA; Figure 4A, lower panel). BFA inhibits the activity of the guanine exchange factor GBF1, thereby inhibiting protein transport from the ER to the Golgi apparatus: on‐going retrograde transport ‘collapses’ the Golgi onto the ER – relocalizing Golgi proteins to the ER. To perform similar protein sub‐cellular localization analyses upon the range of site‐directed mutant forms we created (Figure 3), we needed to introduce a second site mutation (P3A) to ablate the translational recoding activity of the various *Sp*NLR2A6 mutants (Table 1). Analyses of these double mutant forms showed that the mutations upstream of P3A had no discernible effect upon the distribution of mCherry fluorescence within the cell: while some of the mutations we created did affect the translational recoding activity, these mutations did not appear to affect the interaction with SRP (examples shown in Figure 4B).

**Figure 5:**
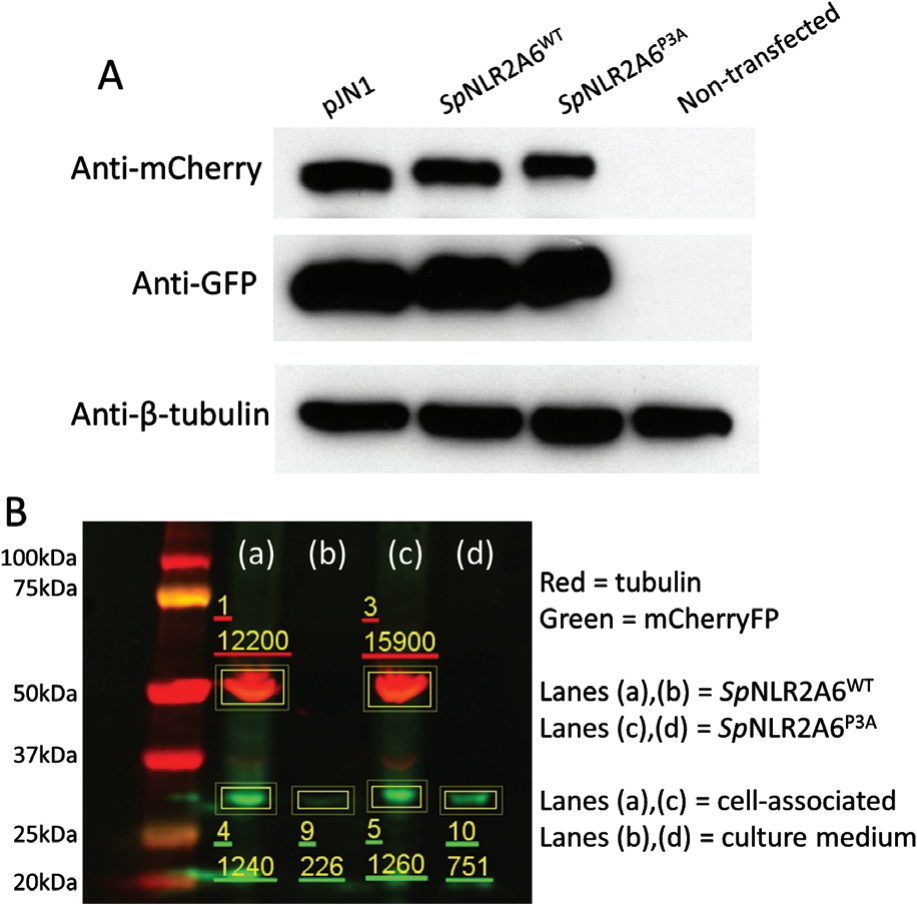
**Analyses of cellular/secreted protein.** A) Western blots from transfected cell lysates showing comparable protein expression levels between pJN1 (encoding [mCherry‐T2A‐GFP]), and pSpNLR2A6 constructs (SpNLR2A6^WT^ ‐mCherry‐T2A‐GFP) encoding either SpNLR2A6^WT^ or SpNLR2A6^P3A^. The negative control comprised non‐transfected HeLa cells. Cells were harvested at 30 h post‐transfection. The cytoskeletal protein β‐tubulin was used as a further positive control. B) Quantitative western blot analyses were performed on cells transfected with pSpNLR2A6^WT^ (lanes a, b) or pSpNLR2A6^P3A^ (lanes c, d). Using Image Studio Lite software, regions of the gel were selected for analyses (boxed areas) and the secondary antibody fluorescent intensity determined for the anti‐β‐tubulin loading controls for cells transfected with pSpNLR2A6^WT^ (lane a, box 1 = 12 200) and pSpNLR2A6^P3A^ (lane c, box 3 = 15 900). Normalizing the mCherryFP fluorescence data using the β‐tubulin loading controls (lane a, box 1 and lane c, box 3) the SpNLR2A6^P3A^ cell‐associated mCherryFP intensity (lane c, box 5 = 1260), becomes 967. In both cases no tubulin was detected in the protein prepared from cell media by (lanes b, d). For cells transfected with pSpNLR2A6^WT^, the proportion of mCherryFP that was cell‐associated (lane a, box 4 = 1240) was 85%, while that in the medium (lane b, box 9 = 226) was 15%. In the case of cells transfected with pSpNLR2A6^P3A^, the proportion of mCherryFP that was cell‐associated (lane c, box 5 = 1260: normalised to 967 using the β‐tubulin loading control) was 56%, while the proportion of mCherryFP in the medium (lane d, box 10 = 751) was 44% – a much higher proportion in the medium.

Transfected cells showed comparable protein expression levels between pJN1 (encoding [mCherry‐T2A‐GFP]), and p*Sp*NLR2A6 constructs (*Sp*NLR2A6*‐*mCherry‐T2A‐GFP) encoding either *Sp*NLR2A6^WT^ or *Sp*NLR2A6^P3A^ (Figure 5, Panel A). Quantitative western blot analyses of the proportions of these translation products which either remained cell‐associated, or, was secreted into the media was performed. For both types of analyses of transfected cells, an anti‐β‐tubulin antibody was used to detect the cytoplasmic protein β‐tubulin as a control. Since this protein is released into the media by cell lysis, the degree of protein ‘cross‐contamination’ between cells and the tissue culture media was performed using both anti‐β‐tubulin and anti‐mCherryFP antibodies. In this manner the mCherryFP within the media (present due to *both* cell lysis plus any secretion) could be directly compared with β‐tubulin within the media (present due to cell lysis *alone*).

Our data showed that in the case of cells transfected with plasmid p*Sp*NLR2A6^P3A^‐mCherry‐T2A‐GFP, significantly more mCherry was detected in the media than that observed for cells transfected with p*Sp*NLR2A6^WT^‐mCherry‐T2A‐GFP (Figure 5, Panel B). The results from the quantitative western blot analyses showed that ∼56% of [*Sp*NLR2A6^P3A^‐mCherry‐T2A] was cell‐associated, with ∼44% present in the media. In the case of the wild‐type *Sp*NLR2A6 sequence, ∼85% of [*Sp*NLR2A6^WT^‐mCherry‐T2A] was cell‐associated, with ∼15% present in the media (Figure 5, Panel B). Interestingly, these data are consistent with the level of *Sp*NLR2A6^WT^ recoding activity which produced approximately the same proportion of *in vitro* translation products with, and without, the N‐terminal *Sp*NLR2A6^WT^ signal sequence (Figure 3). In the case of [*Sp*NLR2A6^P3A^‐mCherry‐T2A], a proportion of this product will naturally be cell‐associated as it traffics through the exocytic pathway. An alternative method of analyzing these transfected cells confirmed these data. Live‐cell imaging using an IncuCyte Zoom microscope (Essen Bioscience) showed the mCherryFP signal to be higher – by the same proportion – for cells transfected with p*Sp*NLR2A6^P3A^ than for cells transfected with p*Sp*NLR2A6^WT^. Here, the cell‐associated mCherryFP fluorescence intensity was normalised against the (cytoplasmically localized) GFP signal. Again, the data are consistent with the secretion of mCherryFP from cells: out of the focal plane and into the media (data not shown).

**Figure 6:**
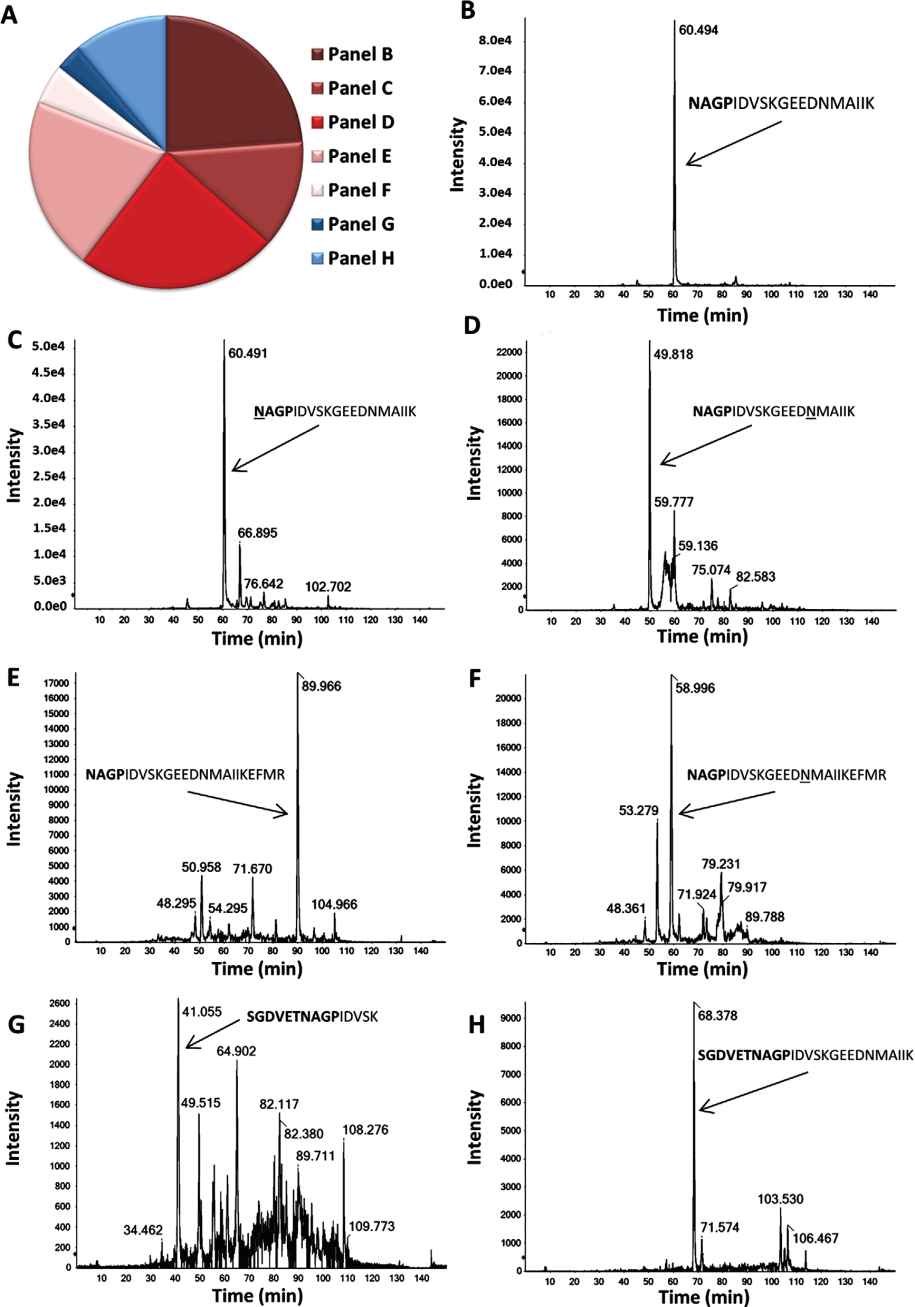
**Mass spectrometry.** Pie‐chart summarizing the relative abundance of each of the SpNLR2A6^P3A^‐derived tryptic peptide species: pink, red etc. shaded areas corresponding to signalase cleaved peptides (B–F), light/dark blue shaded areas corresponding to non‐(signalase) cleaved peptides (G–H) (Panel A). Chart areas correspond to the relative abundance of the individual MS traces obtained from a Coomassie‐stained protein band corresponding to ∼30 kDa (Panels B–H). B–H) show the extracted ion chromatograms (XIC) displaying the extracted peak(s) of the mass to charge ratio (m/z) of the peptide of interest. Plots are time (x‐axis) against intensity (y‐axis). The arrows identify the peak(s) corresponding specific peptide sequence shown. Tryptic peptides detected with N‐termini corresponding to the predicted signalase cleavage site are shown (B–F), together with those uncleaved (by signalase) peptides (G–H). Amino acids in **bold** correspond to C‐terminal residues from the SpNLR2A6^P3A^ peptide, the downstream residues correspond to in‐frame N‐terminal residues from mCherry. Underlined residues have been post‐translationally modified: oxidated (Panels D, F) or deaminated (Panel C).

Soluble transfected‐cell proteins were analyzed by SDS–PAGE and subsequent mass‐spectrometry (Figure 6). Proteins between 25 and 30 kDa were subjected to MS/MS analyses and the data showed that a substantial signal arose from mCherry where the N‐terminal sequence data indicated that the *Sp*NLR2A6^P3A^ (recoding inactive) signal peptide had been ‘cleaved’ away at the site predicted by the SignalP 4.1 algorithm (−VET**^↓^**NAG‐): presumably by the signal peptidase complex.

### Protein localization: expression in plant cells

**Figure 7:**
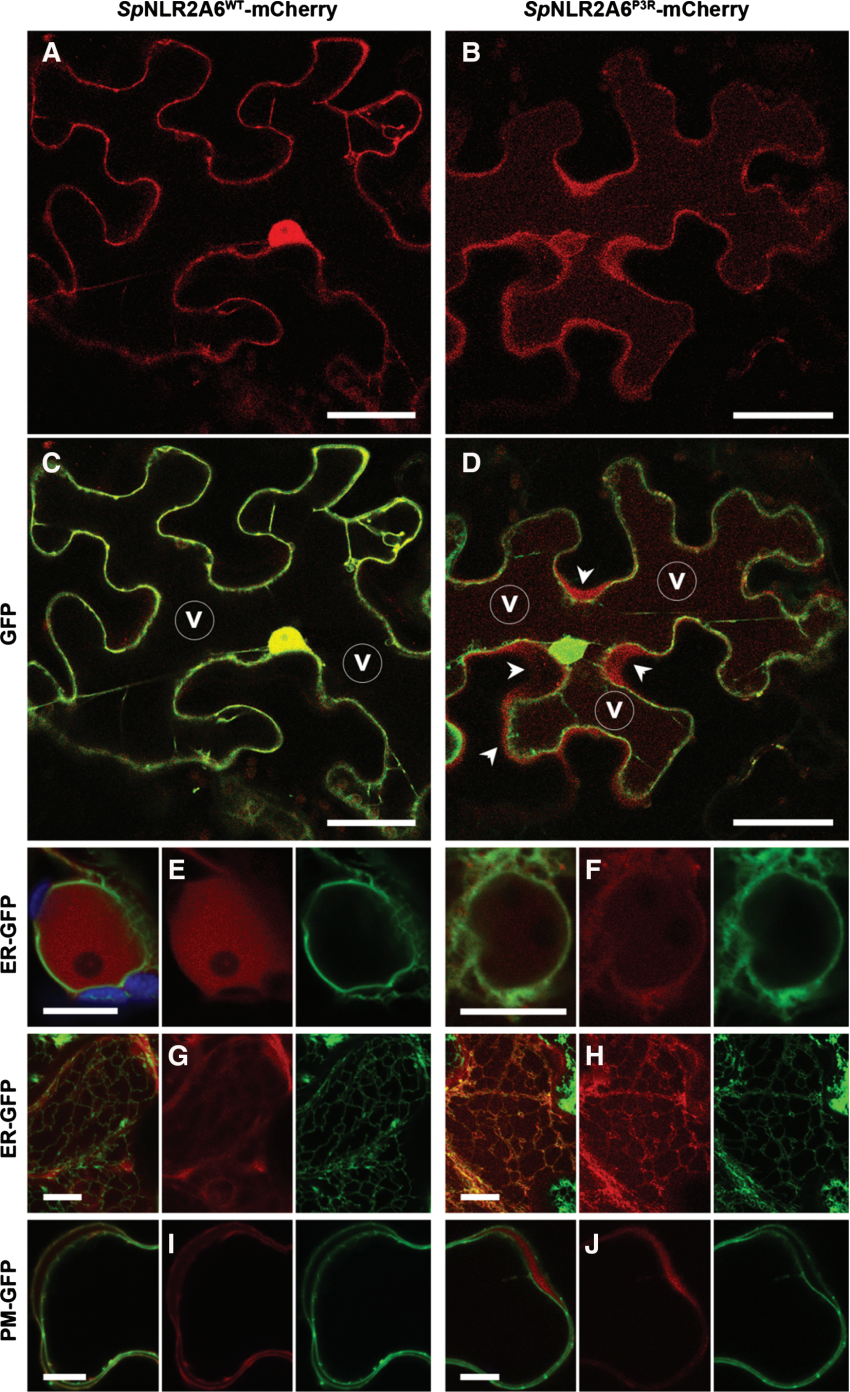
**Expression in planta.** SpNLR2A6^WT^‐mCherry (left column, Panels A, C, E, G, I) and SpNLR2A6^P3A^‐mCherry (right column, Panels B, D, F, H, J) expressed in plant epidermal cells (N. benthamiana). A and C) Expression of SpNLR2A6^WT^‐mCherry and unfused GFP from a SpNLR2A6^WT^‐mCherry‐T2A‐GFP construct. Both fluorescent proteins colocalize in the nucleoplasm and cytosol. B and D) Expression of SpNLR2A6^P3A^‐mCherry and unfused GFP from a SpNLR2A6^P3A^‐mCherry‐T2A‐GFP construct. SpNLR2A6^P3A^‐mCherry fluorescence surrounds the nucleus and is observed in fluorescent halos around the cell (arrow heads in D). Weak fluorescence is also visible in the central vacuole. Left to right panels in (A and B) and (C and D) show mCherry, and mCherry/GFP merged channels, respectively. v: central vacuole. E–H) Expression of SpNLR2A6^WT^‐mCherry and SpNLR2A6^P3A^‐mCherry, respectively, in transgenic plants expressing GFP targeted to the lumen of the ER 34. SpNLR2A6^WT^–mCherry fluorescence localizes to the nucleoplasm, which is surrounded by the GFP‐labelled nuclear envelope (E), and does not colocalize with GFP‐labelled tubular cortical ER (G). By contrast, SpNLR2A6^P3A^‐mCherry fluorescence colocalizes with both the nuclear envelope (F) and cortical ER tubules (H). (I–J) Co‐expression of SpNLR2A6^WT^‐mCherry (I) and SpNLR2A6^P3A^‐mCherry (J), respectively, with GFP targeted to the plasma membrane 31. SpNLR2A6^WT^‐mCherry localizes to the periphery of two adjacent cells whose boundaries are marked by PM‐GFP (I), whereas SpNLR2A6^P3A^‐mCherry localizes to the cell wall space between cells (J). Images are single confocal sections except for panels G, H, which are maximum projections of entire z‐stacks. Scale bars 50 µm (Panels A–D) and 10 µm (Panels E–J).

It should be noted that our analyses of the echinoderm sequences were performed using mammalian cells: for potential biotechnological purposes, we tested the activities of the *Sp*NLR2A6 sequence in plants. When [*Sp*NLR2A6^WT^‐mCherry‐T2A‐GFP] was expressed in *N. benthamiana* leaf epidermal cells, mCherry fluorescence colocalized completely with unfused GFP in the peripheral cytoplasm and nucleoplasm (Figure 7A,C), as is typical of free fluorescent proteins in plants 31. When [*Sp*NLR2A6^P3A^‐mCherry‐T2A‐GFP] was over‐expressed, the resulting mCherry and GFP fluorescence were clearly separated. GFP showed a nucleo‐cytoplasmic distribution with nucleolar exclusion as for [*Sp*NLR2A6^WT^‐mCherry‐T2A‐GFP]. By contrast, mCherry fluorescence weakly labelled the nuclear envelope which is continuous with the ER, indicating that the protein was directed into the lumen of endomembranes by the *Sp*NLR2A6^P3A^ leader (Figure 7B,D). Additionally, in merged mCherry and GFP images, a fluorescent apoplastic halo of mCherry was visible surrounding the GFP‐labelled cytoplasm. This distribution is typical of secreted proteins 29, 32, 33. A very faint mCherry fluorescence was also detectable in the central vacuole; this is sometimes observed with fluorescent proteins directed into the secretory pathway 34, 35 and may be caused by the T2A peptide remaining attached to the C‐terminus of mCherry, as C‐terminal FMDV 2A can act as a vacuolar mis‐targeting signal in *N. benthamiana* epidermis 35. To further verify these observations, we colocalized *Sp*NLR2a6^WT^‐mCherry and *Sp*NLR2A6^P3A^‐mCherry constructs with GFP targeted to the ER lumen 32 and to the plasma membrane 31. The ER marker confirmed the observation that *Sp*NLR2A6^P3A^‐mCherry, but not *Sp*NLR2A6^WT^‐mCherry, colocalized with the nuclear envelope (Figure 7E,F). In cells with high expression levels, *Sp*NLR2A6^P3A^‐mCherry, but not *Sp*NLR2A6^WT^‐mCherry also colocalized with the tubular ER network (Figure 7G,H). Furthermore, using GFP‐labelled plasma membrane as a marker for the cell boundary it was apparent that *Sp*NLR2A6^P3A^‐mCherry, but not *Sp*NLR2A6^WT^‐mCherry, was present in the intercellular cell wall space (Figure 7I,J). Thus, the *Sp*NLR2A6^WT^ sequence mediates cleavage in plants like T2A and FMDV 2A 36, whereas the cleavage‐deficient *Sp*NLR2A6^P3A^ acted as a signal sequence leading to secretion of [mCherry–T2A] into the cell wall.

## Discussion

Dual protein targeting encompasses a range of different mechanisms whereby the translation products arising from a single gene may be distributed across several subcellular sites (reviewed in 37 38). Many of these effects involve post‐translational mechanisms. Proteins may contain a single, ambiguous, signal that may be recognized by more than one type of receptor, each located at a different sub‐cellular site: in plants, the majority of dual‐targeted proteins partition between mitochondria and chloroplasts. Over 100 such dual‐targeted proteins are currently known 39, 40, 41. Proteins may contain multiple, different, targeting signals whose functions may be controlled by accessibility to, and relative affinities for, their respective receptors. Signal sequences may be masked by a range of different mechanisms, or, post‐translation modifications (including proteolysis) may inactivate or completely remove such signals. Mechanisms of dual protein targeting involving co‐translational signal sequences are, however, less common: alternative splicing may produce a mixture of mRNAs – a proportion encoding a signal sequence, a proportion not. In the case of an mRNA that does encode a signal sequence, alternative sites of the initiation of translation produces a mixture of translation products with, and without, signal sequences.

Our data reveals a novel mechanism of dual protein localization by dual‐functional signal sequences; (i) active in translational recoding and (ii) capable of targeting to the exocytic pathway, most likely via an SRP‐dependent process. Our data also shows that relative partitioning of the translation products between the cytoplasm and secretion can be determined by the nature of the translational recoding sequence itself – controlling the levels of translational recoding. It should be noted that the reporter genes we chose for our analyses of SpNLR2A6 do not comprise any other form of localization signal. In natural biological contexts, however, proteins downstream of such signal sequences may well do so. We have postulated that the outcome of translational recoding may be regulated by cellular (translational) stress: that the regulation of the activity of elongation factor 2 (eEF2, proposed to be a key factor in the translation of sequences downstream of 2A) is regulated by eEF2 kinase – which, in turn, is regulated by cellular stress pathways 10, 15. Transposing this model onto the recoding‐type signal sequences reported here, the model would predict that as a response to cellular stress pathways downregulating eEF2 activity, increased stress would lead to decreased synthesis of sequences downstream of such a signal sequence. Alternatively, during evolution, single point mutation of this sequence could regulate the proportions of translation products localizing to the cytoplasm or be secreted from the cell. The manifold biological implications of this system remain to be determined.

With regards the biotechnological utilities which may arise from these observations, the ability to both localize a protein to the cytoplasm and to secrete the same product from the cell has obvious potential applications. 2A has been shown to work in all eukaryotic systems tested to date 19 and the mechanism of protein translocation across the ER membrane is generally conserved across kingdoms 27. With regards our observations using plant cells, 2A peptides have been shown to mediate translational recoding in plants 42, thus the respective functionalities of SpNLR2A6^P3A^ and SpNLR2A6^WT^ in plant cells was not surprising. Since the reporter constructs were delivered by a viral vector (in case of SpNLR2A6‐mCherry‐T2A‐GFP dual reporters) and agrobacteria (in all cases) into plant cells, i.e. in the context of pathogen infections, it might similarly be expected that at least some of the [SpNLR2A6^WT^‐mCherry] was secreted. Indeed, very faint extracellular mCherry fluorescence was observed with this construct (Figure 7I) although the majority of mCherry clearly stayed in the cytoplasm due to translational recoding by SpNLR2A6^WT^.

It is fruitless to speculate as to the evolutionary origins or relationships between cellular (signal sequences/non‐LTR retrotransposons) and virus 2A/2A‐like sequences: given the shortness of these 2A/2A‐like sequences a polyphyletic origin is entirely feasible (even probable), but it is noteworthy that with a relatively modest number of point mutations a ‘classical’ signal sequence could acquire an additional translational recoding capacity – or vice versa!

## Materials and Methods

### Plasmid constructs

Plasmids created for *in vitro* coupled transcription/translation analyses and mammalian cell transfection were based upon a pCDNA3.1 vector backbone, encoding tandem CMV and T7 promoters immediately upstream of the inserts described below. Sites of *Sp*NLR2A6 mutations described below were designated as per the numbering scheme shown in Table 1. All constructs described below were verified by automated DNA sequencing.

**Plasmid p*Sp*NLR2A6^WT^** encodes *Sp*NLR2A6^WT^ fused to GFP (Figure 1C). The plasmid was constructed by PCR amplification of GFP using a forward primer JN1for (5′‐GCTAGCTCTAGAACCATGGATGGATT CTGTCTTCTCTATCTGCTCCTGATCCTCTTGATGAGATCTGGTGACGTTGAAACCAATCCCGGGCCCATCGTGTCCAAAGGGGAA‐3′). This primer encoded *Nhe*I/*Xba*I RE sites (underlined), *Sp*NLR2A6, *Apa*I RE site (underlined: introduced to facilitate subsequent *Sp*NLR2A mutagenesis) plus the sequence encoding the N‐terminal six residues of GFP (omitting the initiating AUG). The reverse primer JN1rev (5′‐CCCGGGATTTTCCTCCAC‐3′) encoded the C‐terminal 6aa of *Thosea asigna* virus 2A (T2A) comprising a *Sma*I RE site (underlined). The PCR product was restricted with *Nhe*I and *Sma*I and ligated into pJN1, similarly restricted.

**Plasmid p*Sp*NLR2A6^P3A^** encodes a mutated – recoding inactive – form of *Sp*NLR2A6 (*Sp*NLR2A6^P3A^) fused to GFP. The plasmid was constructed by PCR amplification of GFP using the forward primer JN2for (5′‐GCTAGCTCTAGAACCATGGATGGATTCTGTCTTCTCTATCTGCTCCTGATCCTCTTGATGAGATCTGGTGACGTTGA AACCAAT***G***CCGGGCCCGGGCCCATCGTGTCCAAAGGGGAA‐3′), identical to JN1for – other than the single point mutation within the *Sp*NLR2A6 (bold, italic, type face), and the reverse primer JN1rev. Cloning into pJN1 was as described above.

**Plasmid pJN1** encodes an [mCherry‐T2A‐GFP] self‐processing polyprotein (Figure 1D). Here, mCherry is linked in a single ORF, *via* a highly active 2A‐like sequence from T2A, to GFP 6. Essentially all the translation products were [mCherry‐T2A] and GFP 8. mCherry was amplified using the forward primer JoN1 (5′‐GGATCC **ATG**GTGAGCAAGGGCGAGGAGGATAA‐3′; *Bam*HI RE site underlined, mCherry initiation codon in bold) and the reverse primer JoN2 (5′‐TCTAGATTTGTACAATTCATCCATGCCG; *Xba*I RE site underlined; mCherry stop codon removed). The PCR product was purified, restricted with *Bam*HI and *Xba*I the ligated into plasmid pJC3 43, similarly restricted. This created a plasmid (pJN5) encoding an [mCherry‐T2A‐mCherry] polyprotein. The mCherryGFP sequences downstream of T2A were then replaced with GFP by amplification of GFP using the forward primer JoN3 (5′‐GGGCCCGATATCGTGTCCAAAGGGGAAG AGCTGTTC‐3′; *Apa*I and *Eco*RV RE sites underlined; GFP initiation codon removed) and the reverse primer JoN4 (5′‐CTCGAGTTACTTATACAGCTCGTCCAT‐3′; *Xho*I RE site underlined). The PCR product was purified, restricted with *Apa*I and *Xho*I and ligated into pJN5, similarly restricted.

**Plasmid p*Sp*NLR2A6^WT^‐mCherry‐T2A‐GFP** encodes a [*Sp*NLR2A6^WT^‐mCherry‐T2A‐GFP] self‐processing polyprotein (Figure 1E). Plasmid pJN1 was restricted with *Pst*I, the 2463 bp fragment (encoding ΔmCherry‐T2A‐GFP) isolated then ligated into plasmid p*Sp*NLR2A6^WT^, similarly restricted.

**Plasmid p*Sp*NLR2A6^P3A^‐mCherry‐T2A‐GFP** encodes a [*Sp*NLR2A6^P3A^‐mCherry‐T2A‐GFP] self‐processing polyprotein. The assembly of this construct was as described for p*Sp*NLR2A6^WT^ above, except the insert was ligated into plasmid p*Sp*NLR2A6^P3A^ restricted with *Pst*I.

### Site‐directed mutagenesis

Point mutations within the *Sp*NLR2A6^WT^ sequence were introduced using the QuikChange system (Agilent) as per the manufacturer's instructions. Primers (Table 2) were obtained from IDT.

**Table 2:**
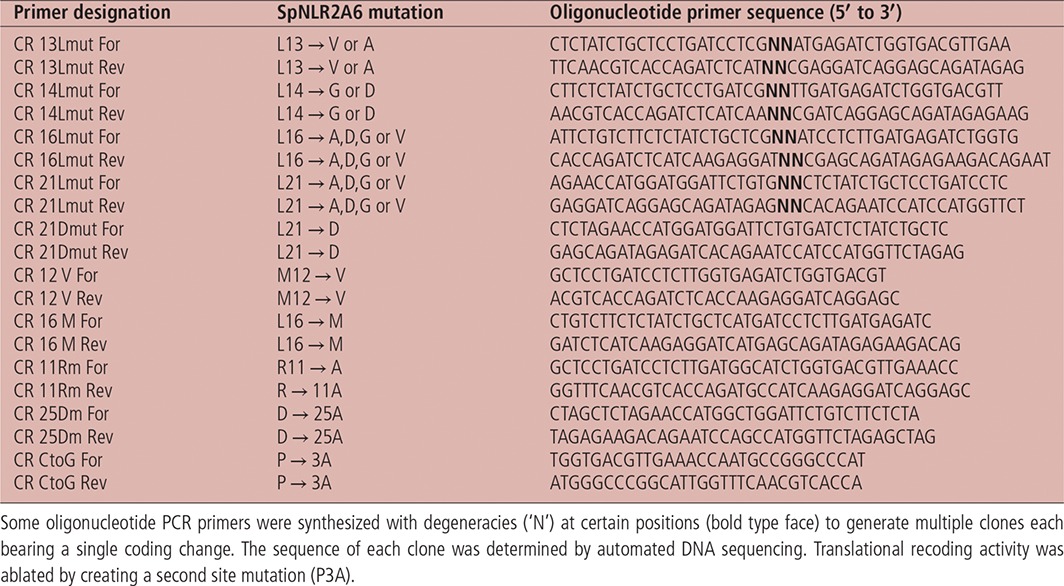
Oligonucleotide primers

### In vitro coupled transcription/translation reactions

To analyze the recoding activity of *Sp*NLR2A sequences, plasmid constructs were used to programme Quick TnT coupled transcription/translation rabbit reticulocyte lysate (RRL) systems (Promega), as described 5. These systems comprise all of the translational apparatus, but the endogenous mRNA (primarily globin mRNA) has been degraded by treatment with micrococcal nuclease, which is then inactivated by chelation of Ca^++^ ions by EGTA. mRNA to program the translation system is generated by T7 RNA polymerase (a supplement to the RRL) driving transcription from the T7 promoter within the plasmid DNA used to program the system. No endomembranes are present unless the reaction mixture is specifically supplemented with microsomes. Briefly, plasmid DNA (100 ng) was used to programme the lysate master mix (10 μL) supplemented with ^35^S‐methionine (10 *μ*Ci). Reactions were incubated at 30°C (90 min) before the addition of 2× SDS–PAGE loading buffer. Translation reactions (5 μL aliquots) were then analyzed using gradient (4–20%) SDS–PAGE gels (Expedeon). Gels were then dried and the distribution of radiolabel determined by autoradiography.

### Transient expression in HeLa cells

HeLa cells were maintained in DMEM supplemented with FCS (10%). For microscopy, cells were transfected once they had reached 50% confluency. 16 h prior to transfection cells were seeded onto 15 mm sterile glass coverslips in 6‐well trays in a final volume of 2 mL culture media. For microscopy, cells were transfected with plasmid DNA (750 ng) and Lipofectamine2000 (Invitrogen; 4 μL) then added to OptiMEM (Life Technologies) to a final volume of 200 μL. Cells were incubated at 37°C for a further 30 h prior to fixation. For western blotting experiments, cells (50% confluency) were transfected with plasmid DNA (1.5 µg) and Lipofectamine2000 (7 μL) then added to OptiMEM to a final volume of 400 μL, cultured at 37°C for a further 30 h prior to harvesting. For brefeldin A (BFA; Sigma‐Aldrich) treatment, BFA was added to a final concentration of 15 µg/mL 45 min prior to fixing.

### HeLa cell microscopy

Cells were fixed using the protocol as described 43. Briefly, the coverslips were washed 2x with PBS, fixed for 10 min in ice‐cold methanol and subsequently washed 2× with deionized water, before mounting on glass microscope slides. Images were obtained using a Deltavision microscope and images analyzed using the resolve 3d software package.

### Cell extracts

Extracts were prepared using the protocol as described 43. Briefly, 30 h post‐transfection media was removed and cells washed 2× with 1 mL of PBS, harvested in 1 mL PBS pelleted by centrifugation (100g, 5 min). The cell pellet was resuspended in 70 μL RIPA buffer (NaCl, 150 mm; Tris pH 7.4, 10 mm; Triton X‐100, 1%, Na deoxycholate 1% and SDS, 0.1%). EDTA‐free protease inhibitor cocktail was added freshly (1:20 v/v), the cells incubated on ice for 30 min and centrifuged at 3540g (4°C, 20 min). The cellular debris pellet was discarded and the supernatant analyzed by 12% SDS–PAGE.

### Western blotting

Proteins were transferred to a nitrocellulose membrane using an iBlot system as per the manufacturer's instructions (Life Technologies). Membranes were then blocked in PBST with non‐fat dried milk (5%) for 1 h prior to probing with anti‐mCherry, anti‐GFP or anti‐β‐tubulin primary antibodies overnight. Post incubation, membranes were washed 3x in PBST. Bound antibodies were detected using HRP‐secondary antibody (Dako) in PBST with milk (1 h). Membranes were then washed 3× in PBST, rinsed in deionized water and finally subjected to enhanced chemi‐luminescence by incubation in freshly‐prepared visualization solution (2 min). The membrane was exposed to autoradiography film (Kodak) for 3 to 45 seconds, as appropriate.

### Quantitative western blotting

Cell extracts were prepared as described above. Cell culture media were clarified by centrifugation (4°C, 500 × ***g***, 10 min), then four volumes of acetone (pre‐chilled to −20°C) were added, vortexed for 20 seconds and incubated at −20°C for I h. The protein suspension was pelleted (4°C, 15 000 × ***g***, 10 min), the supernatant carefully removed and the pellet air‐dried for 30 min prior to being dissolved in SDS gel loading buffer. Equal proportions of proteins derived from either the total cell extract or cell medium were subjected to SDS (10%) gel electrophoresis then transferred to Immobilon‐FL membranes using the wet transfer method (Merck‐Millipore). Membranes were blocked using Li‐Cor blocking buffer (PBS), probed using anti‐mCherry or anti‐β‐tubulin primary antibodies as described above, then with Li‐cor IRdye 800CW and IRdye 680RD secondary antibodies. Membranes were then analyzed using a Li‐Cor Odyssey scanner and the images analyzed using Image Studio Lite. For either p*Sp*NLR2A6^WT^‐mCherry‐T2A‐GFP or p*Sp*NLR2A6^P3A^‐mCherry‐T2A‐GFP plasmid transfected cells, the proportion of mCherryFP that was either cell‐associated or in the culture media was calculated by dividing the intensity of each fluorescent intensity signal (from the secondary antibody) by the combined total.

### Mass spectrometry

The region of a Coomassie‐blue stained SDS gel corresponding to 25–30 kDa was excised and cut into 1 mm cubes which were then subjected to in‐gel digestion using a ProGest Investigator in‐gel digestion robot (Genomic Solutions) using standard protocols 44. Briefly, the gel cubes were de‐stained by washing with acetonitrile and subjected to reduction and alkylation before digestion with trypsin at 37°C. Peptides were extracted with 10% formic acid and concentrated down to 20 μL using a SpeedVac (ThermoSavant). Peptides were then separated using a nanoLC Ultra 2D plus loading pump and nanoLC AS‐2 autosampler equipped with a nanoflex cHiPLC chip based chromatography system (Eskigent), using a ChromXP C18‐CL trap and column (Eskigent). Peptides were eluted with a gradient of increasing acetonitrile, containing 0.1% formic acid (5–25% acetonitrile in 75 min, 25–80% in a further 15 min). The eluent was sprayed into a TripleTOF 5600 electrospray tandem mass spectrometer (ABSciex) and analyzed in Information Dependent Acquisition (IDA) mode, performing 250 milliseconds of MS followed by 100 millisecods MSMS analyses on the 20 most intense peaks determined by MS. The MS/MS data file generated was analyzed using the ProteinPilot 4.1 Paragon algorithm (ABSciex) against an internal protein database to which the [*Sp*NLR2A6^WT^‐mCherry‐T2A] peptide sequence had been added, with trypsin as the cleavage enzyme and carbamidomethyl modification of cysteines. The Mascot algorithm (Matrix Science) was set for trypsin cleavage at only one end of the peptide (semi‐trypsin), carbamidomethyl as a fixed modification of cysteines and methionine oxidation plus deamidation of glutamines and asparagines as variable modifications.

### Plant expression vectors

*Sp*NLR2A6^WT^‐mCherry‐T2A‐GFP and *Sp*NLR2A6^P3A^‐mCherry‐T2A‐GFP reporter constructs were amplified from p*Sp*NLR2A6^WT^‐mCherry‐T2A‐GFP and p*Sp*NLR2A6^P3A^‐mCherry‐T2A‐GFP, respectively, using the forward primer 5′‐ATAGCGTTAATTAAAGCTCTAGAACC**ATG**GATGGATTCTG‐3′ and reverse primer 5′‐A**TTA**ATGCGGCCGCCTCGAGTTACTTATACAG‐3′ (*Pac*I / *Not*I restriction sites underlined; translation start/stop codons in bold typeface). PCR products were digested with *Pac*I and *Not*I and ligated into the TMV‐based over‐expression vector pTRBO, similarly restricted 30. For colocalization with GFP organelle markers, *Sp*NLR2A6^WT^‐mCherry and *Sp*NLR2A6^P3A^‐mCherry were excized as *Xho*I‐*Not*I fragments from plasmids p*Sp*NLR2A6^WT^ and p*Sp*NLR2A6^P3A^, respectively, blunt‐ended with T4 DNA polymerase and ligated into *Xmn*I/*Eco*RV‐linearized pENTR1A (Invitrogen). The resulting Gateway^®^ entry vectors were recombined with the plant expression vector pGWB402Ω 45.

### Plant inoculations

pTRBO.*Sp*NLR2A6^WT^‐mCherry‐T2A‐GFP and pTRBO.*Sp*NLR2A6^P3A^‐mCherry‐T2A‐GFP were each electroporated into *Agrobacterium tumefaciens* (strain AGL1) cells. Single agrobacteria colonies were grown in liquid culture at 28°C for 2 days, pelleted and resuspended in infiltration medium (10 mm MES; 10 mm MgCl_2_; 15 µm acetosyringone) to an optical density of 0.001 at 600 nm. The agrobacterium suspension was infiltrated into small incisions on the abaxial side of *N. benthamiana* leaves using a syringe without a needle. pGWB402Ω‐based expression vectors and the GFP‐LTI plasma membrane marker 31 were similarly introduced by agrobacteria infiltration, except that the suspension had an optical density of 0.25 at 600 nm. For colocalization with the ER, pGWB402Ω constructs were agroinfiltrated into transgenic *N. benthamiana* plants expressing ER‐targeted GFP 32. Plants were kept at 25°C, 16 h light/8 h dark and imaged at 4 days post‐infiltration.

### Plant cell imaging and image analysis

Infiltrated leaves were detached and adhered onto microscope slides with the abaxial side facing up using double‐sided sticky tape. Leaves were imaged on an upright SP2 confocal laser scanning microscope (Leica) equipped with a 40× water dipping lens. GFP was excited at 488 nm and detected at 495–525 nm, mCherry was excited at 594 nm and detected at 600–630 nm. Images were exported into imageJ software for ratiometric measurements and into adobe photoshop for assembly of figures.
